# Palliative care in small-scale living facilities: a scoping review

**DOI:** 10.1186/s12877-024-05259-9

**Published:** 2024-08-24

**Authors:** Brittany S. DeGraves, Judith M. M. Meijers, Carole A. Estabrooks, Hilde Verbeek

**Affiliations:** 1 Faculty of Nursing, University of Alberta, Edmonton, AB Canada; 2 Department of Health Services Research, Care and Public Health Research Institute, Maastricht University, Maastricht, Netherlands; 3 Living Lab in Ageing and Long-Term Care, Maastricht, Netherlands

**Keywords:** Small-scale living, Group home, End of life, End of life care, Palliative care, Dementia, Nursing home, Scoping review

## Abstract

**Background:**

Innovative small-scale facilities for dementia focus on providing quality of life and maintaining the functional abilities of residents while offering residents a home for life. To fulfill the home-for-life principle, palliative care approaches are necessary to maintain quality of life in these facilities. Few studies have reported on how palliative care is provided to residents in small-scale facilities. The aim of our review is to determine the extent to which palliative care approaches are reported in small-scale facilities.

**Methods:**

A scoping review of the literature using recommended methods from the Joanna Briggs Institute. Four databases, CINAHL, PubMed, PsycINFO, and Web of Science, were searched for studies published from 1995 to 2023. One reviewer completed the title, abstract and full-text screening and data extraction; two additional team members piloted the screening and extraction process and met with the main reviewer to make decisions about article inclusion and ensure consistency and accuracy in the review process. The extracted data was open-coded and analyzed using thematic analysis. The data was then synthesized into themes using palliative care domains for dementia.

**Results:**

Of the 800 articles obtained in the search, only ten met the inclusion criteria: six from Japan, two from the Netherlands, and one each from Austria and the United States. In most small-scale facilities, palliative care is important, with facilities prioritizing family involvement and person-centred care, minimizing resident discomfort and enhancing residents’ remaining abilities until the end of life. The included studies did not discuss palliative care policies or professional staff training in depth.

**Conclusions:**

This study provides an overview of the literature on palliative care in small-scale facilities for individuals with dementia. Most facilities focus on residents’ wishes at the end of life to enhance comfort and provide a home-like environment. However, more research is needed to further understand the quality of palliative care approaches in these homes.

**Supplementary Information:**

The online version contains supplementary material available at 10.1186/s12877-024-05259-9.

## Background

Innovative small-scale care environments are designed to meet the complex needs of residents with dementia by maintaining their functional ability and quality of life in a person-centred way while maintaining a true home-like setting [1–3]. Small-scale facilities provide physical and psychosocial care for older adults or individuals who are no longer able to live independently. Small-scale facilities differ from long-term care facilities because they have a smaller number of residents (six to fifteen on average). These facilities provide a community environment and person-centred care while stimulating resident autonomy by promoting active participation in social activities, household chores, and decision-making surrounding daily schedules [1–5]. To create a home-like environment, these facilities include home-like kitchens, living rooms, and private bedrooms while also allowing residents to bring pets and furniture from home [1, 3, 6]. Staff in these environments are often responsible for multiple additional tasks, including cooking and cleaning [3]. Small-scale facilities are also “a home for life”, meaning that residents can stay and live a purposeful life within these homes until their death [3, 7].

There are multiple types of small-scale facilities internationally [3]. These include Group Living/Homes (Japan/Sweden), Small-scale Living Facilities (Netherlands/Belgium), Green Care Farms (Netherlands), Residential Groups (Germany), Cantou (France), Care Housing (Scotland), Domuses (United Kingdom), Woodside Place (Canada/United States), and Green Houses (United States) [3, 8]. Recently, research has focused on the outcomes of living in small-scale homes compared to traditional long-term care; however, few studies have reported on palliative care approaches in these homes [3–5, 9]. With these studies reporting that residents in small-scale homes have increased social and physical engagement and functioning compared to residents in long-term care and with these facilities growing in popularity, it is critical to understand if these facilities can provide palliative care and a home for life for residents with dementia. Therefore, factors surrounding palliative care in these innovative facilities, including the quality of palliative care and what can be improved, need to be identified [1, 10].

To live within these small-scale settings until death, a palliative approach is essential, as it promotes a focus on person-centred, family-centred and holistic care while trying to improve the quality of dying [11, 12]. Palliative care is also essential for providing residents with peace at the end of life, as only half of individuals with dementia in long-term care die peacefully, according to their families [13]. The quality of care that residents receive toward the end of life is also a more relevant care goal than increasing the length of residents’ lives due to the complicated nature and symptom burden associated with dementia [12, 14, 15].

No review has yet to be completed on the topic of palliative care in small-scale facilities. Our purpose in conducting a scoping review was to identify the current body of knowledge and to identify research gaps in this field. Our research question was “To what extent are palliative care approaches reported in small-scale home-like facilities internationally?”

## Methods

We conducted a scoping review to identify the available literature and determine how palliative care approaches are used in small-scale facilities [16, 17]. Our search strategy and review were based on the framework proposed by the Joanna Briggs Institute Reviewers Manual for scoping reviews [17]. This framework proposes enhancements made to Arksey and O’Malley’s framework [16]. We followed the recommended steps of (1) defining the objectives and research questions, (2) developing inclusion criteria and aligning them with the research question, (3) describing a planned approach to searching, data extraction and presentation, (4) searching for evidence, (5) selecting the studies or evidence, (6) data extraction, (7) analysis of data and evidence, (8) presentation of results, and (9) connecting the evidence to the purpose of the review and stating the implications of the findings [17].

### Search strategy and study identification

To identify keywords and create a search string for our literature search, Medline, Google Scholar, and CINAHL were searched to analyze the text words and abstracts of the retrieved papers. A health sciences librarian, in addition to the writers, helped define terminology and add to the search strategy. The final search used all identified terms in the steps described in Table 1.

Index terms were constructed once the search strategy was finalized in all the following databases: CINAHL, PubMed, PsycINFO, and Web of Science. Our literature search covered from 1995 onward; for the full search strategy, please see Additional File 1, PubMed Final Search Strategy. The decision to search from 1995 onward was made in partnership with research team members and a health sciences librarian who had familiarity with the literature and discussed this as an adequate timeline to explore both critical concepts identified in our study (palliative care for individuals with dementia, and small-scale dementia care facilities). We also completed an ancestry search of the reference lists of the retrieved papers. We conducted a grey literature search using the search engine Google Scholar. Our initial search was conducted in July 2019 and was updated in September 2023.

**Table 1:** Detailed search terms based on the research question and query

Step	Search terms
1	*Subject Area 1: Small-scale living facilities* Green Care*, Care farm*, small scale living, small scale*, Group home, homelike*, homelike care environments, shared housing arrangements, shared hous*, green house, homes for the aged, social farming, multifunctional agriculture, farming for health, group living, collective living, group dwelling, small units, special care*, residential groups, CADE units, Cantou, Care Housing, Domus philosophy
2	*Subject Area 2: Palliative care* Palliative Care, Palliative*, end of life*, death, hospice, hospice care, good death, palliative medicine, terminal care, terminal*, hospice and palliative care nursing, attitude to death, death education, hospice patients, death attitudes
3	*Subject Area 3: Dementia* Dementia*, Dementia patient*, geriatric*, gerontologic care, gerontologic nursing
4	*All Subject Areas*

We also consulted six experts in palliative care and small-scale living home-like facilities in multiple countries (North America and Europe) to determine if they had access to additional literature focused on this subject area; four answered our inquiry.

### Selection criteria

#### Inclusion criteria

Inclusion criteria were: (1) studies must have collected primary data; (2) the study aims had to have explicitly addressed small-scale residential models (settings that resemble home-like environments, including having a maximum of 15 residents per house or unit, having staff, residents, and their family form a household together sharing responsibilities, staff performing integrated tasks both medical and personal [3, 5]); (3) studies must have described palliative care or end-of-life care approaches; (4) study samples had to include adults aged 65 years or older; (5) studies must have focused primarily on residents with dementia; and (6) studies must have been in English, Dutch, or German languages.

#### Exclusion criterion

Studies could not have been conducted in a hospice or traditional long-term care facility.

### Study selection

Before conducting the review, the selection criteria and review processes were discussed until a consensus was reached between BD, HV, and JM. The selection criteria were piloted in title/abstract and full-text screenings by BD, HV and JM to ensure the criteria were straightforward and to determine agreement between team members. Based on the selection criteria, one reviewer (BD) screened all titles, abstracts, and full-text articles. Two additional team members, HV and JM, each conducted title/abstract and full-text screenings on a subsection of the retrieved articles to ensure consistency and accuracy with BD’s screening decisions. HV and JM also reviewed BD’s screening decisions and notes throughout the screening process. During the screening process, articles that did not directly match the criteria or were deemed questionable by BD were reviewed individually by the three reviewers, who then met to determine the eligibility and make decisions about article inclusion. During these meetings, all decisions and questions that arose during the screening process were discussed with the team to ensure transparency and consistency and reach a consensus. Each full-text article was also discussed in meetings between the three team members (BD, HV, and JM), and an agreement was reached on which papers were to be included.

### Database management

We exported the retrieved studies to Endnote X9 for management and deduplication. The review update was conducted using Covidence. Thematic analysis was performed with the assistance of NVIVO-12, which we used to open-code the extracted data and cluster the data into themes.

### Data extraction

The extraction process was piloted by three reviewers (BD, HV, JM). Data from the retrieved articles were extracted and summarized by BD, and the extraction tables were reviewed for accuracy and consistency by two team members (JM and HV). We extracted the following information from each study: (1) title of the literature, (2) study design, (3) research question, (4) country of data collection, (5) study sample, (6) type of housing, (6) results and discussion, and (7) study limitations.

### Analysis and synthesis of results

The extracted data were synthesized using thematic analysis, where the author read and became familiar with the data, began to open-code included studies and grouped these codes into sub-themes. The subthemes were then clustered into themes using van der Steen et al.’s [15] domains of palliative care [18]. These domains of palliative care received international consensus from experts in multiple countries in a European Association for Palliative Care (EAPC) whitepaper to promote high-quality care for individuals with dementia, as there are no preexisting guidelines for end-of-life care for individuals with dementia [15]. For an in-depth overview of van der Steen et al.’s [15]. palliative care domains for dementia, please see Table 2.

**Table 2:** van der Steen et al.’s domains of palliative care [15]

Domain	Definition
1. Applicability of palliative care	The appropriateness and application of a palliative care approach for residents with dementia.
2. Person-centred care, communication and shared decision-making	Following each person until death and having resident wishes as their number one priority. Including the family and residents in care decision-making.
3. Setting care goals and advanceplanning	Use of proactive advance care planning, such as living wills, that should start as soon as the diagnosis is made. Actively involving residents in end-of-life decision making and revisiting care planning regularly with the resident and family.
4. Continuity of care	Ensuring continuity of care even on patient transfer and between all disciplines through appropriate communication on care plans.
5. Prognostication and timely recognition of dying	Prognostication and mortality cannot be predicted accurately; however, staff should use clinical judgement and resources to indicate the time of death with an accurate assessment to notice changes in the resident condition.
6. Avoiding overly aggressive, burdensome, or futile treatment	Evaluating medications and transfers of patient care to the hospital by assessing risks and benefits, as well as the resident’s care goals.
7. Optimal treatment of symptoms and providing comfort	Using a holistic approach to care, using tools to assess pain and behaviour, and evaluating the effectiveness of interventions. Using both nonpharmacological and pharmacological treatment for physical symptoms as needed.
8. Psychosocial and spiritual support	Promoting a comfortable environment up until death, including assessment of spiritual needs and providing sources of support such as religious rituals even in advanced dementia. Providing both the resident and family with emotional support.
9. Family care and involvement	Providing families with support throughout the dementia trajectory, educating them about dementia and palliative care and encouraging family involvement in care.
10. Education of the healthcare team	Education in palliative care for dementia should be provided to all healthcare team members, including volunteers on all domains listed (1–9).
11. Societal and ethical issues	Patients with dementia should have access to palliative care, awareness for palliative care for dementia is needed and collaboration between palliative and dementia care should be promoted.

The data were reported based on the 2018 PRISMA Preferred Reporting Items for Systematic Reviews and Meta-Analyses extension for Scoping Reviews (PRISMA-ScR) Checklist [19]. This was performed by BD and checked by JM and HV for clarity and to reach a consensus on the data charting and extraction methods.

## Results

**Fig. 1:**
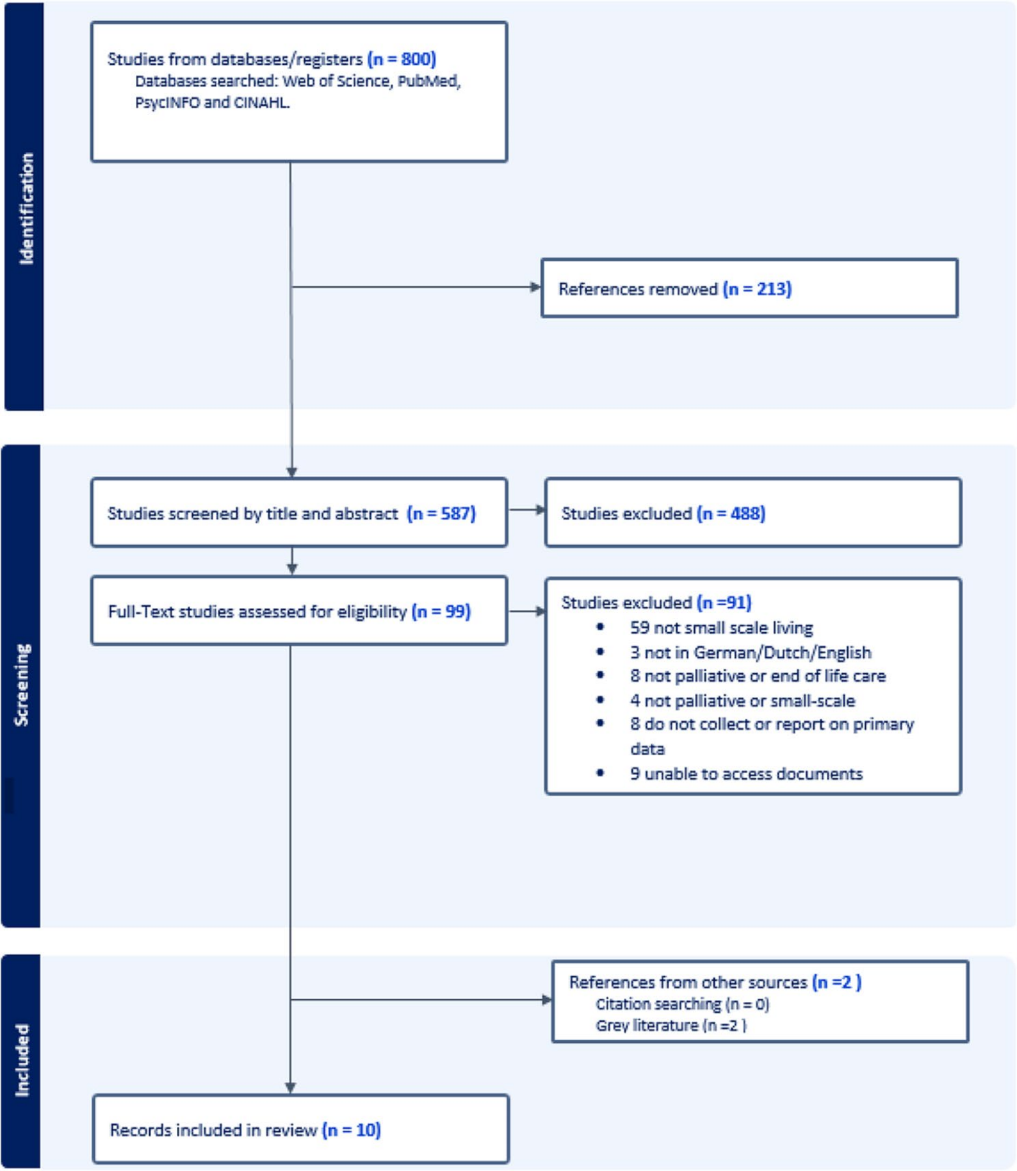
PRISMA flow diagram [20]

Our search retrieved a total of 800 references from PsycINFO, PubMed, CINAHL, and Web of Science databases; 213 duplicates were removed. After title and abstract screening, 99 articles met our criteria for full-text review. Figure 1 shows the PRISMA diagram [20]. Our final sample of ten reports included eight papers from the initial search and two records (one policy document and one unpublished dataset) from the grey literature.

The articles included in the scoping review were from Japan (6), Austria (1), the Netherlands (2), and the United States (1). Nine papers were in English, and one was in German [21]. The article in German [21] was initially translated by Google Translate, and the translation was reviewed by a research associate fluent in German. Of the ten papers, six focused on group homes (GH) in Japan, [2, 22–26] which house 5–9 residents with mild to moderate dementia and encourage residents to participate in activities of daily living in a home-like setting [2, 3]. The concept of group homes in Japan is similar to other homes included in this study, including small-scale facilities in the Netherlands, shared housing (WG’s) in Austria, and Green Houses in the United States, which also have shared home-like kitchen and living areas and private bedrooms and recommend participation in daily activities [3]. For the characteristics of the included reports, please see Table 3.

**Table 3:** Characteristics of the selected studies

Study and Year	Country	Research Objective	Design and Methods	Sample	Facility Type	Study Limitations
1: Anbäcken et al. (2015) [22]	Japan	To focus on the expressions of identity of residents with dementia at a Japanese group home.	Qualitative exploratory study	19 residents	Group Home	Only includes one care home and has a relatively small sample.
			Participant observations and interviews with six residents.			
2: Hirakawa et al. (2006) [23]	Japan	To clarify current end-of-life policies and practices of group homes in Japan.	Survey	1689 managing directors of group homes and policy items from 1639 care homes.	Group Home	The response rate was not satisfactory, as group homes with regressive policies did not respond. Due to the number of group homes doubling between the time the study was conducted and the publication, results may not accurately reflect the group home situation at the time of publication.
			Self-reported questionnaire about group homes, end-of-life policies, and end-of-life experiences.			
3: Kobayashi et al. (2008) [2]	Japan	To explore the components of end-of-life care provided to residents with dementia in group homes.	Qualitative exploratory	7 group home administrators who have provided end-of-life care to a resident.	Group Home	The limited number of participants who were not randomly selected (snowball sampling) therefore limited generalizability. No other perspectives outside of administrators’ views were explored.
			Exploratory open-ended interviews were analyzed with a constant comparative approach.			
4: Nakanishi and Honda (2009) [24]	Japan	Clarify processes of decision-making and end-of-life care for residents with dementia in group homes.	Retrospective cross-sectional	33 cases of end-of-life care from 17 group homes across Japan.	Group Home	The retrospective study could result in selection bias, the small sample may not be generalizable, and it was not possible to collect information on quality evaluations of end-of-life care in the group homes.
			Self-administered questionnaires completed by managing directors.			
5: Nakanishi et al. (2016) [25]	Japan	Examine the association between care quality for residents with dementia and professional caregivers’ perspectives regarding palliative care for dementia.	Cross-sectional.	2116 professional caregivers from 329 agencies (217 home long-term care support, 29 small-scale/multiple home care providers, and 83 group homes).	Group Home	Due to the cross-sectional design causal analysis cannot be made. Low response rate and sampling bias possible.
			Questionnaires, collected data about approximately 3603 people diagnosed with dementia and the quality-of-care measures used over four weeks.			
6: Reitinger et al. (2010) [21]	Austria	To address where and how people who live in shared housing die. In addition to how end-of-life care is dealt with and if the care provided is influenced by hospice and palliative care principles.	Qualitative exploratory approach	33 people (22 women 11 men), including management and employees of the residential communities, experts in palliative care, relatives of deceased individuals, and doctors with experience in these care homes.	WGs (outpatient residential communities, shared housing) - Wohlfarts and welfare mix	Qualitative interviews with only 33 people at one point in time.
			Interviews with different stakeholder groups from this field. Three focus groups and an expert discussion were used to supplement the interviews.			
7: Smit et al. (2022) [27]	Netherlands	To explore experiences of family caregivers and healthcare workers with end-of-life care for people with dementia who died on a green care farm.	Qualitative, descriptive and explorative design with a phenomenological approach.	15 participants (7 healthcare workers and 8 family caregivers).	Green Care Farms	Findings may not be generalizable, and there is potential for selection bias as only a small number of green care farms were included.
			Semi-structured interviews focused on end-of-life care, COVID-19 and bereavement support.			
8: Takada et al. (2022) [26]	Japan	To develop an inventory to assess the implementation of advanced care planning for people with dementia in group homes in Japan. And examine the association between the implementation of an advanced care planning inventory and the quality of dying.	Cross-Sectional survey	569 surveys from managers and care planners from 2000 group homes across Japan.	Group Homes	There was a low response rate to the survey, with responses potentially coming only from facilities that had a positive experience in end-of-life care, and the study only included homes that provided end-of-life care. Surveys only looked at the point of view of the care worker, which may be different than the views of family members or individuals living with dementia.
			Mailed surveys (2 types) of care workers in group homes in Japan – 1 focused on managers and 1 focused on care planners for residents who passed in the facility. Each survey focused on the experience of a single resident who had passed in the facility.			
						Items related to the survey looking at the facility staff and family members were removed from the distribution of the survey. Therefore, only the component of advanced care planning looking at residents was addressed.
			Analysis: Logistic regression models. Dependent variable: Quality of Dying in Long Term Care (QOD-LTC) scale. Independent variables: The advanced care planning practice inventory (ACP-PI) ran with covariates. Higher scores of QOD-LTC indicate a higher quality of end-of-life care. Higher scores on items in the ACP-PI indicate a higher level of implementation of ACP components.			
9: The Green House Project (2012) [28]	United States	Grey Literature. Values document describing values and practices to be used at all Green House homes across the United States.	Policy and values document.	N/A	Green House	Values and practices document that does not describe whether or not these values are followed at all green house homes.
10: van der Steen (2016) [29]	Netherlands	Unpublished Dutch End-of-Life in Dementia (DEOLD) dataset. Focuses on the importance of person-centered small-scale environment on dying.	Unpublished Dutch End of Life in Dementia (DEOLD) data. Uses the End of life in Dementia Satisfaction with Care and Comfort Assessment in Dying tools to measure the quality of dying.	*N* = 330	Small-scale living	Unpublished data.

### Study characteristics and design

Seven of the final ten reports had the primary goal of investigating palliative care in dementia [2, 21, 23–25, 27, 29]. Two studies [22, 28] had a more general aim in which palliative care was only mentioned briefly. The last study, Takada et al. (2022) [26] aimed to develop an advanced care planning inventory and compare this inventory to the quality of death of residents with dementia.

Four studies used qualitative techniques and explored more detailed descriptions of palliative care approaches in small-scale homes [2, 21, 22, 27]. Five studies used quantitative assessments to compare palliative care practices among a larger population [23–26, 29]. One article focused on examining palliative care practices in one home [28].

Below, we summarize findings based on van der Steen et al.’s domains of palliative care [15]. An in-depth summary of the thematic analysis results is provided in Table 4.

**Table 4:** Thematic analysis: results of each reviewed study as applied to the palliative care domains [15]

Domain of palliative care	Studies which addressed this theme/domain	Study findings
Domain 1: Applicability of palliative care	1. Anbäcken et al. (2015) [22]	Life is lived fully until death.
	2. Hirakawa et al. (2006) [23]	16.9% of GH’s provided palliative care. 47.2% would provide it under certain conditions. 27.5% will not offer end-of-life care, though 76.4% state it was possible if necessary.
	3. Kobayashi et al. (2008) [2]	Maximizing quality of life and preparation are key in end-of-life care. Four essential care components: (1) maintaining a familiar lifestyle, (2) minimizing discomfort, (3) proactively using medical care, (4) family involvement.
	6. Reitinger et al. (2010) [21]	Residents stay in the shared flats until the end of life. The last phase of life in these homes is comparable to hospice care.
	9. The Green House Project (2013) [28]	The end-of-life care process is inclusive and reflects upon palliative and hospice principles. The older adult will remain in this home until the end of life.
Domain 2: Person-centred care	1. Anbäcken et al. (2015) [22]	Resident council meetings are held to discuss end-of-life, and residents are seen as capable of discussing death.
	2. Hirakawa et al. (2006) [23]	65.5% of staff asked residents their wishes for end-of-life care, and 68% reported asking family their wishes.
	3. Kobayashi et al. (2008) [2]	The priority of staff is residents’ wishes and needs. Meeting the extra needs of residents while minimizing the risk requires extra attention and care, which administrators and staff go out of their way to give.
	6. Reitinger et al. (2010) [21]	End-of-life care is tailored to residents’ wishes and needs. Wishes of all parties are also considered.
	7. Smit et al. (2022) [27]	The residents’ individual needs and preferences are the primary focus in the green care farms, with importance placed on knowing the residents well and learning their life stories. The environment of the care farm created a homelike atmosphere, with the residents’ requests being listened to and adhered to. Staff felt they were able to take more time with residents on care farms compared to other settings (i.e., nursing homes).
	8. Takada et al. (2022) [26]	There is a significant association (*p* < 0.001) between the Quality of Dying-LTC (QOD-LTC) scale scores and the ACP-PI (Advanced Care Planning Practice Inventory) factor “Provision of information and conversation with the resident to encourage them to express their end-of-life wish” (when all three factors of the ACP-PI were run simultaneously in the logistic regression model). This factor was more significantly related to QOD-LTC outcomes than other ACP-PI factors, including items such as providing information about end-of-life care and discussing end-of-life factors, indicating the necessity to share knowledge and hold conversations with each resident surrounding end-of-life care. When run individually in the regression model, the ACP-PI component “Devising to encourage the resident to express their wishes with consideration with dementia” was also found to have a significant association with quality of death (QOD-LTC); this factor involves providing an individual with dementia with the ability to express their intentions in their end-of-life period.
Domain 3: Setting care goals	2. Hirakawa et al. (2006) [23]	Homes with progressive care policies were more likely to have experience and training with palliative care and be affiliated with long-term care and hospitals. 65.5% of staff asked residents their wishes for end-of-life care, and 68% asked the family.
	3. Kobayashi et al. (2008) [2]	Resident wishes at end-of-life are a priority. Prior preparation for end-of-life care services is recommended as it allows for the administration to be prepared for the deterioration of the resident.
	4. Nakanishi and Honda (2009) [24]	60.6% of managing directors played a central role in end-of-life planning. Residents’ wish of place of death was confirmed in 18.2% of cases. 90% of family members wished the place of death to be a GH, but nearly half of residents wanted to die at home.
	6. Reitinger et al. (2010) [21]	Living wills are written in some facilities as early as possible, while in other facilities, living wills are only used as a guideline (or safeguard) for staff or are not needed at all.
	8. Takada et al. (2022) [26]	The composite score and each factor score of the ACP-PI (the advanced care planning practice inventory) for individuals with dementia were found to have a significant association (*p* < 0.001) with the quality of death-long-term care (QOD-LTC) in a logistic regression analysis. Indicating that the individuals living in the group homes who had passed away had an association between a positive increase in their quality of death (measured by QOD-LTC) and an increase in the use of advanced care planning (as measured by ACP-PI). The three factors of ACP-PI that were also associated with a significant increase in QOD-LTC scores (higher quality of end-of-life care) included: 1) Provision of information and conversation with the resident to encourage them to express their end-of-life wishes 2) Preparations in case the resident becomes unable to express their own end-of-life wishes- this includes advanced care planning measures such as designating an alternate decision maker or writing down one’s intentions, such as in a living will. 3) Devising to encourage the residents to express their wishes with consideration for their dementia - this factor includes including practices according to the level of cognitive functioning experienced by the person with dementia and providing support for the person with dementia to be able to express their intentions.
	9. The Green House Project (2013) [28]	Older adults are seen to have direct involvement in their care plan, according to the essential practices of these homes.
Domain 4: Continuity of care	2. Hirakawa et al. (2006) [23]	Progressive policy homes were less likely to work with in-home services but were more confident in providing onsite care and grief support and were more likely to work with hospitals.
	3. Kobayashi et al. (2008) [2]	Staff closely observe and record residents’ condition to provide others with more information. Group homes must have a collaborative relationship with a medical institution.
	7. Smit et al. (2022) [27]	Support of multiple disciplines was identified as a subtheme in the analysis; this includes close contact between healthcare workers, physicians, and psychologists during the end of life phase. Workers also suggested that a shared guideline for end-of-life care for all healthcare workers from various disciplines to refer to would be helpful.
Domain 5: Prognostication and timely recognition of dying	3. Kobayashi et al. (2008) [2]	Accurate assessment and close observation are used to notice even the slightest changes in the resident nearing the end-of-life period.
	4. Nakanishi and Honda (2009) [24]	GH staff were able to anticipate the time of death, and unexpected changes in the residents’ condition were not usual.
	7. Smit et al. (2022) [27]	The dying phase is typically short at green care farms, which may be due to the environment that encourages activity and a short time spent in bed before death, for example, only two days. Some deaths are, therefore, very sudden and catch family and healthcare workers by surprise.
Domain 6: Avoiding overly aggressive, burdensome, or futile treatment	2. Hirakawa et al. (2006) [23]	Both regressive and progressive policy group homes have equal relations with hospitals. Progressive homes were more likely to have arrangements with hospitals for end-of-life care services. 72.9% of GH responded that a designated hospital could administer end-of-life support if necessary.
	3. Kobayashi et al. (2008) [2]	Hospital outpatient departments are used for rapid deterioration, and home care nurses help manage symptoms. Staff recognized the lack of dignity in hospitals and how residents are often confined to wheelchairs, left unattended, and progress in their dementia when in hospital.
	4. Nakanishi and Honda (2009) [24]	Residents require additional care at the end of life, including diapers for bowel movements and urination, the use of IV feeding one week before death, and residents becoming dependent on personal hygiene. The amount of care from staff increases one week before the end of life.
	5. Nakanishi et al. (2016) [25]	GH residents were treated with more physical restraint and antipsychotic use than in-home long-term care support. Physical restraints and antipsychotic use were found to be used regardless of professional caregivers’ knowledge and attitudes regarding end-of-life care.
	6. Reitinger et al. (2010) [21]	Hospitals were seen as not suitable for people with dementia, and typically, residents, staff, and families worked together to avoid hospital stays or keep them as short as possible.
Domain 7: Optimal treatment of symptoms and providing comfort	2. Hirakawa et al. (2006) [23]	The following comfort measures were reported (% GH): massage (39.6%), touch (59.1%), and support of comfortable posture (61.5%).
	3. Kobayashi et al. (2008) [2]	Minimizing physical and mental discomfort is essential in end-of-life. Staff compensate for decreased functioning, for example, by providing elaborate assistance for impaired mobility.
	5. Nakanishi et al. (2016) [25]	GH residents were older and had greater activities of daily living impairment than residents treated by in-home long-term care or multiple home care providers.
	7. Smit et al. (2022) [27]	A primary theme in the analysis was compassionate care and support in the dying phase. This included an emphasis on comfort being the primary focus at the end-of-life phase, including the provision of compassionate care and potentially palliative sedation to prevent burdensome symptoms (i.e., pain). This focus on comfort helped family caregivers to feel comfortable and accept their relative’s death.
	10. van der Steen (2016) [29]	Small-scale homes show no difference in comfort during end-of-life compared to long-term care homes. However, small-scale homes are associated with better quality of dying, especially personhood.
Domain 8: Psychosocial and spiritual support	1. Anbäcken et al. (2015) [22]	Staff members are at the resident’s bedside during passing.
	2. Hirakawa et al. (2006) [23]	48.4% of GH report providing a good environment at the end of life. Many supports are provided, including grief support (69%), active listening (51%), and getting close (60.9%). Support, including religious healing and advice about property law, were less often available.
	3. Kobayashi et al. (2008) [2]	Despite worsening confusion, staff tried to maintain a familiar lifestyle for residents to reduce anxiety and improve comfort. This was managed by encouraging interaction with residents, providing favorite meals, and having residents sit at their daily spots, even at the end of life.
	4. Nakanishi and Honda (2009) [24]	Deaths occurred under the supervision of care workers (87.9%), attending doctors (69.7%), management directors (63.6%), nursing staff (60.6%), and family members (57.6%).
	5. Nakanishi et al. (2016) [25]	Scores for interactions with surroundings were higher in residents in group homes relative to those for other patients, and scores for self-expression closer to the end-of-life were significantly higher in those treated in group homes than in other patients.
	6. Reitinger et al. (2010) [21]	Dying individuals receive special attention with time for conversation and feelings. All people in the community can actively participate in everyday life; as dementia progresses, social activity decreases. Group activities are almost not possible at end-of-life.
Domain 9: Family involvement	1. Anbäcken et al. (2015) [22]	The family is involved in planning farewell ceremonies based on cultural practices.
	2. Hirakawa et al. (2006) [23]	6.1% of GH report advising about law and property management. Only 37.2% responded they supply users and families with information on the type of end-of-life care available at their homes. GH report providing various forms of grief support, including letters, phone calls, and overall grief care for the family.
	3. Kobayashi et al. (2008) [2]	Collaborating with family is essential in end-of-life care. Staff aim to improve the environment to allow for more family involvement and promote participation in care, for example, by encouraging the family to sleep in the same room and feed the resident. Changes in the resident’s condition were reported to the family to help them make informed decisions.
	4. Nakanishi and Honda (2009) [24]	Approximately half of the residents were attended to by family members who stayed in Group Homes. Only 57.6% of deaths occurred under the supervision of family members.
	6. Reitinger et al. (2010) [21]	Family members often feel like they are in charge of decisions of care, which counteracts the ideas of staff; this is why communication is required for good end-of-life care. The residents stay in their rooms after passing so roommates, employees, and relatives have time to say goodbye.
	7. Smit et al. (2022) [27]	Family caregivers are actively involved in the resident’s care from admission to the end of life and are invited to actively participate in care and provide opinions. Family and care workers are considered as a team and optimize care together, i.e., family are allowed to sleep at the farm when needed. Mutual communication was positive between both parties with regular phone calls, text messages with photos and in-person visits. This allows the family to be informed and involved in every decision and to check in on family members’ feelings. Regular conversations, even before the dying phase begins, promote an effective relationship. In some homes, family caregivers receive wake baskets with educational information.
	9. The Green House Project (2013) [28]	Family involvement is valued in care planning and life events.
Domain 10: Education of healthcare team	2. Hirakawa et al. (2006) [23]	40.4% of group homes report providing staff education about end-of-life care. The most common topics are focused on the etiology and symptoms at the end of life, physical care, mental support for family and residents, and palliative care communication skills.
	3. Kobayashi et al. (2008) [2]	Administrators created manuals on what to do during end-of-life care and developed educational strategies and mental health support for staff while increasing the number of staff working during the end-of-life. Staff training to improve their observation skills regarding end-of-life was provided as end-of-life care unfolded. Staff also learned from their experiences of assessing residents.
	5. Nakanishi et al. (2016) [25]	Knowledge of and attitudes toward practicing palliative care were higher in group homes than in in-home long-term care support and small-scale multiple-home providers. Quality of life and exhibition of minimum negative behaviour were significantly higher when caregivers exemplified knowledge and positive attitudes toward palliative care.
	6. Reitinger et al. (2010) [21]	Competent around-the-clock care and support are regarded by nursing staff as important in providing end-of-life care. Hospice or palliative care courses are now becoming more common.
	7. Smit et al. (2022) [27]	Family caregivers and healthcare workers interviewed emphasized that the knowledge surrounding end-of-life care differs within the team of workers. They described how both nurses and nurse assistants have valuable knowledge and experience to contribute to the care team.
Domain 11: Societal and ethical issues	2. Hirakawa et al. (2006) [23]	Many group homes report that they only provide end-of-life care under the following conditions: (1) no medical intervention, (2) an understanding on the part of users (residents) and families of the limits and abilities of the group home, (3) understanding of the staff of the limits and abilities of the group home, (4) no complaint of pain.
	3. Kobayashi et al. (2008) [2]	Each administrator gives end-of-life care because they want each resident to have a humane end-of-life period despite financial and staffing restrictions that make group homes not typically equipped to provide this care.
	7. Smit et al. (2022) [27]	COVID-19 provided a potential barrier for green care farms to provide end-of-life care as they had to adhere to specific national guidelines; however, despite guidelines, care workers emphasized the need for family caregivers to say goodbye properly. Visits were decreased during COVID-19; however, adaptations were made, including visiting from a distance using window visits. There were barriers to end-of-life care, including personal protective equipment, which made communicating with the person with dementia difficult.
	8. Takada et al. (2022) [26]	The home receiving an end-of-life care bonus (provided to homes to incentivize quality care and advanced care planning at the end of life) was significantly associated with the QOD-LTC scale outcomes in the logistic regression. Particularly associated with an increase in QOD-LTC scale scores.

*Notes: GH*: Group Homes, *GCF*: Green Care Farm, *QOD-LTC*: Quality of Dying Long-Term Care (higher scores indicate higher quality of end-of-life care), *ACP-PI:* (Advanced Care Planning Practice Inventory)

### Domain 1: applicability of palliative care

Palliative care services are important in small-scale facilities due to residents’ increasing age and their dementia diagnosis [21, 28]. Maintaining a familiar lifestyle, minimizing discomfort, proactively utilizing medical care, and collaborating with family members are essential to end-of-life care [2].

Hirakawa et al. (2006) [23] state that despite most group homes reporting that it was possible to provide palliative care (76.4%), 27.5% of these homes would not provide this care, and only 22.3% provided end-of-life care.

### Domain 2: person-centred care, communication, and shared decision-making

Having the residents’ wishes as the first priority and including residents in care was discussed in six studies. For example, asking residents’ wishes for end-of-life and incorporating wishes into regular routines, such as asking about preferences for meals, music, and activities [2, 21–23, 27]. In some group homes, residents regularly discussed end-of-life wishes (i.e., location of death) with other residents during regular resident council meetings [22]. Takada et al. (2022) [26] also identified a significant relationship between having conversations with residents to express end-of-life wishes (ACP-PI component) and improved quality of end-of-care (increases in the QOD-LTC scores) of individuals who had passed away in group homes in Japan.

### Domain 3: advanced care planning and care goals

Managing directors (60.6%) were the primary palliative care planners in group homes and were responsible for confirming resident wishes and directing care, such as organizing staff training to prepare for resident deterioration [2, 24]. In Reitinger et al.’s (2010) study, homes described contradictory views on living wills (advanced directives). Some homes preferred living wills to be written as soon as possible (i.e., upon admission), while other homes only used them as a safeguard for the staff or did not require them at all [21].

Hirakawa et al. (2006) [23] reported that 68% of staff members asked families about their wishes for end-of-life care, and 65.5% of staff members asked residents about their wishes. Residents with dementia in many homes were viewed as capable of discussing end-of-life wishes and care planning [2, 22, 28]. Similarly, Takada et al. (2022) [26] emphasized the significance of advanced care planning for residents with dementia, as they reported that the advanced care planning practice inventory (ACP-PI) had a significant association (*p* < 0.001) with improvements in the quality of end of life care (QOD-LTC) of residents who had passed away in group homes.

Identifying and respecting residents’ wishes at the end of life did not occur in all group homes; for example, in Nakanishi and Honda’s (2009) [24] study of 33 end-of-life cases, residents’ preferred place of death was confirmed in only 18.2% of cases.

### Domain 4: continuity of care

Group homes and green care farms aim to maintain optimal continuity of care by having staff closely observe and record resident conditions to provide detailed information to other healthcare professionals [2, 23, 27]. These collaborations included close contact between healthcare workers, physicians and psychologists. Healthcare workers shared how a guideline for end-of-life care within the home for all disciplines would be beneficial [27]. Group homes also report collaborative relationships with medical institutions, which are often mandatory; however, Hirakawa et al. (2006) [23] report that group homes with progressive end-of-life care policies were less likely to have these affiliations with in-home institutions. Managers in group homes set criteria for physicians to follow during end-of-life consultations and treatment. If the criteria were not met, physicians were replaced [2, 23].

Reitinger et al. (2010) [21] report avoiding or shortening hospitalizations of residents at the end of life with methods including encouraging residents, families, and staff to work together to maintain continuity of care by avoiding hospitalizations. This was found to be an important component of care, as hospitals were identified to not be suitable for residents with dementia as they were often left unattended and lacking in basic dignity while in this setting [2, 21]. However, group homes in Japan associated themselves with hospitals to provide end-of-life or emergent care [2, 23].

None of the 10 studies reported how the continuity of care between facilities was maintained, especially at the end of life, nor did they report barriers to care or care transfers.

### Domain 5: prognostication and timely recognition of dying

Staff reported anticipating resident death through accurate assessments, such as recognizing that a decline in the residents’ appetite and changes in activities of daily living including how becoming dependent on personal hygiene signals the beginning of the end-of-life period [2, 24]. Smit et al. (2022) also described how the end-of-life phase is often short in green care farms, possibly due to the encouragement of activity up until death, and this sometimes results in deaths that are sudden and may come as a surprise to staff and family members [27].

### Domain 6: avoiding overly aggressive, burdensome, or futile treatment

Avoiding aggressive treatment is achieved by carefully examining therapeutic measures; for example, care homes try to avoid hospitalization due to these environments, often leading to poor outcomes [2, 21].

However, Nakanishi et al. (2016) [25] report more frequent use of physical restraint and antipsychotics with residents in Japanese group homes than in those receiving home care (in-home long-term care support or multiple care providers). These treatments were used regardless of the professionals’ attitudes and knowledge regarding palliative and dementia care. The authors do not identify in their results why group homes have more aggressive treatments compared to other community providers [25].

### Domain 7: optimal treatment of symptoms and providing comfort

Contrary to the findings of Nakanishi et al. (2016) [25], minimizing mental and physical discomfort is essential in end-of-life care. In many cases, staff compensate for decreased residents’ ability to maintain quality of life, such as assisting with impaired mobility to enhance residents’ remaining abilities [2]. Staff use many methods to provide physical and psychological comfort, such as supporting a comfortable posture, touch, and massage while providing optimal treatment for discomfort and pain [2, 23, 27].

However, in van der Steen’s (2016) [29] unpublished data from the Dutch End of Life in Dementia (DEOLD) study of 330 care homes, small-scale facilities exhibited no difference in comfort at dying but a better quality of dying compared to traditional long-term care.

### Domain 8: psychosocial and spiritual support

Group homes provide residents with opportunities for discussion of feelings and provision of grief support near the end of life. These homes also promote a familiar lifestyle and comfort by encouraging interactions with other residents and maintaining familiar relationships, as this helps decrease agitation and promotes quality of life in the end-of-life period [2, 21, 23]. Similarly, Nakanishi et al. (2016) [25] found that compared to in-home and multiple small-scale home providers, residents in group homes had higher scores of interactions with their surroundings and self-expression closer to the end of life.

Group homes also report a smaller opportunity to heal religiously and provide education about property law within their facilities [23].

### Domain 9: family care and involvement

Family involvement is encouraged by staff by providing the family with a bed to stay at the residents’ bedside at the end of life and encouraging the family to participate in care, such as by picking out residents’ clothes [2, 27]. Family education also includes palliative care services and objective health changes experienced by residents to help families make end-of-life care decisions [2, 23, 27]. However, Hirakawa et al. (2006) [23] found that only 37.2% of group homes within their study provided families with information about palliative care services in their homes.

Most homes focused on family involvement in medical treatment and end-of-life decision-making, including asking about wishes for their loved one’s care. These discussions were held on multiple occasions, including at the beginning of the resident’s stay and as the resident’s condition deteriorated [2, 24, 27]. In some homes, the managing director handled discrepancies between residents’ and families’ wishes [2, 24].

In many group homes, aftercare rituals and grief support for staff and families are important in honouring the resident and supporting the community after the resident’s death [21, 22]. These rituals include planning ceremonies with families and maintaining cultural practices to honour the resident’s relationships with the family, staff, and residents [22]. Group homes with more progressive policies on palliative care also reported providing more grief support to families. In contrast, only 69% of group homes reported providing this via various methods, such as phone calls [23].

### Domain 10: education of the healthcare team

Nursing staff regard competence in providing care support as critical for providing end-of-life care [21]. Staff also describe feeling anxious about providing care when they lack detailed knowledge about the decline of the resident’s condition [2]. Education surrounding palliative care in group homes includes various topics, such as living wills, communication skills, mental support for residents and family, and end-of-life symptoms and care, to adequately prepare staff to provide palliative care services [23]. These educational resources have become more common in small-scale homes and are provided via various methods, such as manuals, case studies, or work experience. Teaching sessions also provide staff members with mental health support surrounding their positions as palliative care providers [2, 21, 23].

Increases in quality of life, self-expression, interaction with the surrounding environment, and exhibition of minimum negative behaviour in residents are associated with caregivers having greater knowledge and positive attitudes toward palliative and dementia care [25].

### Domain 11: societal and ethical issues

Kobayashi et al. (2008) [2] describe that despite financial and staffing restrictions from the Japanese long-term care insurance system that result in these facilities being ill equipped to provide palliative care, administrators choose to provide this care to maintain residents’ quality of life until death. However, many group homes in Hirakawa et al. (2006) report that homes will only provide palliative care when the resident requires no medical intervention, there is no complaint of pain from the resident, and when there is an understanding by all parties surrounding the limitations to care that the group home can provide [23].

Takada et al. (2022) [26] was the only study to briefly address national strategies or incentives to provide palliative care. They discussed an end-of-life care bonus, which incentivized nursing homes and group homes to improve the quality of care and advanced care planning at the end of life. In particular, this study revealed that homes that received this national incentive had a significant association with higher QOD-LTC scores and, therefore, higher quality end-of-life care.

Smit et al. (2022) [27] discuss the implications of COVID-19 for end-of-life care, particularly how staff and caregivers identified barriers to providing care due to specific national guidelines implemented during the pandemic.

No papers discuss the training of caregivers during their professional education domain [15].

## Discussion

### Main findings

To our knowledge, this scoping review is the first to provide an overview of palliative care approaches in innovative small-scale dementia care homes. We found few studies discussing palliative care in small-scale facilities; however, the included studies identified that it is possible to provide palliative care approaches and a “home for life” for residents with dementia in these innovative facilities.

The findings in the 10 included articles suggest that it is possible to provide palliative care for individuals with dementia within innovative small-scale facilities. The homes in this review prioritized resident and family-centred palliative care approaches while providing a familiar environment for residents, avoiding hospital admissions, and providing quality psychosocial and physical care up until death [2, 21–23, 25, 27]. By maintaining these priorities, these small-scale homes are prioritizing a “home-for-life” principle, which involves maintaining an environment that provides comprehensive care to residents with dementia and a place where they can live and maintain their quality of life up until death [3, 7, 27, 30]. The home-for-life principle and quality palliative care approaches in these homes support residents’ and families’ wishes when they need it the most [11, 12].

### Barriers to palliative care

While our results suggest that palliative care is possible in these homes, the included studies rarely discussed barriers to palliative care. Identifying and considering how barriers may influence the ability to provide quality palliative care in these settings is important. One particular barrier identified was the COVID-19 pandemic, which influenced the ability of staff to maintain resident comfort, particularly due to visitation restrictions for family caregivers [27]. Additional barriers to providing palliative care for dementia in *traditional* long-term care homes include time commitment, lack of equipment, and inadequate staffing [31]. Additional research is needed to identify the barriers and potential solutions in innovative small-scale facilities, including barriers newly introduced by the COVID-19 pandemic.

### Palliative care approaches in small-scale facilities internationally

The papers in this review touched on all general themes of van der Steen et al.’s [15] palliative care domains for dementia and applied them to palliative care within these innovative homes. Most of the papers in this review were from Japan. Therefore, our results must be interpreted with some caution because they are likely influenced by cultural factors specific to Japanese group homes. This includes factors such as the end-of-life bonus implemented in Japan in 2006 to care homes (including group homes) to incentivize the quality of palliative care and advanced care planning in care homes toward the end of life [26, 32]. Studies in this review, such as Hirakawa et al. (2006) [23], were published in the early years of this program, and the group homes included in this paper were the only small-scale facilities in our review with strong relationships with hospitals. These relationships may provide additional care resources and relate to Japan’s national strategy for dementia, which emphasizes avoiding hospitalizations of residents by using a trained outreach team to treat individuals with dementia in the community [2, 23, 24, 32, 33]. This is particularly important for Japanese group homes, as not all group homes are constructed to provide end-of-life care and are not required to have trained medical professionals on site; this differs from other small-scale homes, such as green houses in the United States, which hold hospice principles and have some specialized palliative care training [21, 24, 28]. Avoiding hospitalizations is a common theme in many homes included in this review. This may be due to the association of hospital use with poor outcomes for residents with dementia, including aggressive treatments, functional decline, falls, and dehydration [2, 21, 34, 35]. This is consistent with our findings that many small-scale homes prioritized similar palliative care domains, such as reducing discomfort [15].

Another example of the influence of culture and end-of-life care policies on end-of-life care is how resident autonomy and advanced care planning are treated near the end of life. Hirakawa [36] describes that resident autonomy is a key factor in the cultural difference between Japan and Western Countries, as Western Countries have high respect for patient autonomy, while in Japan, when an individual lacks decision-making capacity, both the family and physicians play an important role in decision making. Hirakawa [36] also describes how patients in Japan often have submissive attitudes toward medical professionals concerning their concept of life and death. Similarly, Groenewoud et al. [37] discuss how Japanese individuals were less likely than Dutch individuals in their study to want to discuss end-of-life care in advance, and more Japanese individuals reported that they would be a burden to family members if they were dependent on help at the end of life. Nakanishi et al. [33] also explored the differences between end-of-life care policies for dementia in multiple countries using van der Steen et al.’s [15] recommendations for palliative care for dementia. Nakanishi et al. [33] found that palliative care policies vary significantly among countries, including the Netherlands, Japan, and the United States, on topics such as the applicability of palliative care, staff education, and person-centred care. This finding is consistent with that of Krikorian et al. [38], who report that ideas about a good death or end-of-life care relate to financial issues, religion, disease, and cultural factors. Despite the cultural factors that may have influenced our findings, many components of palliative care remain consistent despite cultural and geographical contexts, particularly pain and symptom control [39]. While the studies included in the review did not identify the cultural or contextual implications influencing palliative care, this research gap should be addressed in the future.

### Palliative care domains for dementia

Some of van der Steen et al.’s [15] recommendations were covered to a greater extent in some of the papers included in this review. The studies included in this review did not adequately describe all palliative care components. This may be the case, as many of the studies included did not aim to describe palliative care in terms of the van der Steen et al. [15] domains. Palliative care domains such as advanced care planning, palliative care policies, and palliative care education require more research to determine how these processes occur in small-scale facilities. This is important as a focus on palliative care and improving palliative care education is needed both within small-scale facilities and at the public policy level to improve care at the end of life for individuals with dementia and reduce the emotional distress of family and staff members [40, 41].

### Strengths & limitations

*Strengths.* We used a rigorous review approach, including using the Joanna Briggs Institute Reviewers Manual for a scoping review, to structure our search and review process [17]. The van der Steen et al.’s domains for palliative care with dementia, which we used to interpret our findings, have received international consensus from experts in multiple countries [15]. Using these domains, we identified key gaps surrounding what is currently known about palliative care approaches in these small-scale dementia care facilities.

*Limitations.* Only papers written in English, German, or Dutch were included in this study, which may limit the number of relevant papers included in the review. The included studies were from four high-income countries, with the majority from Japan. Thus, this paper may not account for the variation in small-scale facilities in other countries. Differences in culture, national policies, and facilities, such as access to resources, may also influence the palliative care approaches of various small-scale facilities, limiting our findings’ generalizability. For example, while there are similarities between small-scale homes, there are differences in terms of access to outdoor space, participation in group activities, staffing, and the inclusion of cultural rituals that may affect palliative care approaches in these facilities [3, 22]. The decision to have a single reviewer conduct most of our article screenings may have influenced the sensitivity of our screening process and the number of papers included in our review. However, we aimed to reduce that risk by (1) using strict selection criteria, (2) piloting our selection criteria, and (3) having two additional reviewers conduct subsections of the screening process and reviewing the screening notes and decisions made by the primary reviewer (BD).

### Implications

This review identifies the current knowledge base and practices used in small-scale innovative facilities to maintain quality of life and end-of-life for residents living with dementia. The findings of our review can help inform future research in this area by identifying research gaps surrounding palliative care in these innovative facilities. Specifically, future research is needed to identify the following:

- Outcomes of palliative care approaches in small-scale facilities compared to traditional homes.
- Differences and similarities between countries and cultures regarding palliative care in small-scale facilities.
- Various outcomes and practices related to palliative care in different models of small-scale living facilities (comparative evaluation). This includes research on the quality of palliative care, barriers to palliative care, advanced care planning, policies, and education.

## Conclusions

This scoping review is the first of which we are aware that attempts to characterize palliative care in small-scale innovative dementia care facilities. Our results suggest that it is possible to provide palliative care and a “home for life” for individuals with dementia in these settings. This paper offers a glimpse into the palliative care approaches in these innovative facilities, particularly their focus on minimizing discomfort and prioritizing patient and family-centred care at the end of life. Most studies in this area have focused primarily on Japanese Group Homes; therefore, cultural factors may have influenced our results. Future research is, therefore, needed to understand the palliative care approaches in small-scale homes internationally and how these innovative facilities compare to traditional long-term care homes.

### Electronic supplementary material

Below is the link to the electronic supplementary material.

Supplementary Material 1

## Data Availability

No datasets were generated or analysed during the current study.

## Abbreviations

ACP-PI: Advanced care planning practice inventory
CINAHL: Cumulative index to nursing and allied health literature
DEOLD: Dutch end-of-life in dementia
EAPC: European association for palliative care
GCF: Green care farm
GH: Group home (Japan)
PRISMA: Preferred reporting items for systematic reviews and meta-analysis
QOD-LTC: Quality of dying long-term care scale
WG: Wolfarts and welfare mix (Austria shared housing)
