# Supplementary material for: Palliative care in small-scale living facilities: a scoping review
Source: BMC Geriatr. 2024 Aug 24;24:700. doi: 10.1186/s12877-024-05259-9 (PMC11344427; doi:10.1186/s12877-024-05259-9)
Supplement: Supplementary file 1 — Supplementary Material 1 [file 12877_2024_5259_MOESM1_ESM.pdf]

Additional File 1

PubMed Final Search Strategy

**Block 1**	Green Care* [tiab] OR Care farm* [tiab] OR small scale living*[tiab] OR small scale*[tiab] OR Group homes [MeSH] OR Group Home*[tiab] OR homelike*[tiab] OR homelike care environments[tiab] OR shared housing arrangements [tiab] OR shared hous*[tiab] OR green house [tiab] OR homes for the aged [MeSH] OR social farming [tiab] OR multifunctional agriculture [tiab] OR farming for health[tiab] OR group living [tiab] OR collective living[tiab] OR group dwelling [tiab] OR small units[tiab] OR special care* [tiab] OR residential groups [tiab] OR CADE units [tiab] OR Cantou [tiab] OR Care Housing [tiab] OR domus philosophy [tiab]
**Block 2**	Palliative Care [MeSH] OR Palliative care [tiab] OR Palliative*[tiab] OR end of life*[tiab] OR death [MeSH] OR death [tiab] OR hospice*[tiab] OR hospice care [MeSH] OR hospice care [tiab] OR good death [tiab] OR palliative medicine [MeSH] OR terminal care [MeSH] OR terminal care [tiab] OR terminal*[tiab] OR hospice and palliative care nursing [MeSH] OR hospice and palliative care nursing[tiab] OR Attitude to Death [tiab] OR Death education [tiab] OR Hospice Patients [tiab] OR death attitudes [tiab]
**Block 3**	Dementia[MeSH] OR Dementia*[tiab] OR Dementia Patient* [tiab] or Geriatric* [tiab] OR Gerontologic Care [tiab] OR Gerontologic Nursing [tiab
